# Post-Translational Modifications in Atopic Dermatitis: Current Research and Clinical Relevance

**DOI:** 10.3389/fcell.2022.942838

**Published:** 2022-07-07

**Authors:** Xin Ma, Yi Ru, Ying Luo, Le Kuai, Qi-Long Chen, Yun Bai, Ye-Qiang Liu, Jia Chen, Yue Luo, Jian-Kun Song, Mi Zhou, Bin Li

**Affiliations:** ^1^ Department of Dermatology, Yueyang Hospital of Integrated Traditional Chinese and Western Medicine, Shanghai University of Traditional Chinese Medicine, Shanghai, China; ^2^ Shanghai Skin Disease Hospital of Tongji University, Shanghai, China; ^3^ Institute of Dermatology, Shanghai Academy of Traditional Chinese Medicine, Shanghai, China

**Keywords:** atopic dermatitis, post-translational modifications, skin disorders, therapeutic potential, epigenetics

## Abstract

Atopic dermatitis (AD) is a chronic and relapsing cutaneous disorder characterized by compromised immune system, excessive inflammation, and skin barrier disruption. Post-translational modifications (PTMs) are covalent and enzymatic modifications of proteins after their translation, which have been reported to play roles in inflammatory and allergic diseases. However, less attention has been paid to the effect of PTMs on AD. This review summarized the knowledge of six major classes (including phosphorylation, acetylation, ubiquitination, SUMOylation, glycosylation, o-glycosylation, and glycation) of PTMs in AD pathogenesis and discussed the opportunities for disease management.

## Introduction

Atopic dermatitis (AD) is one of the most common heterogeneous diseases, affecting 2.7%–20.1% of children and 2.1%–4.9% of adults worldwide (Barbarot et al., 2018; Silverberg et al., 2021). It manifests as systemic inflammation and epidermal barrier disruption, with multifactorial genetics, age, ethnicity, and geography (Yew et al., 2019). Ameliorating clinical signs and allergy burdens, preventing of recurrence and comorbidities, and improving quality of life are the key points on the disease management. Meanwhile, the allergic symptoms of AD are varied and complex, which range from mild to severe and even life-threatening anaphylaxis.

Extrinsic environmental factors are responsible for tremendous impacts on allergic disease; besides, the epigenetic modification is thought to determine, at least partly, in etiology and pathophysiology of allergies (Alashkar Alhamwe et al., 2020). Studies based on quantitative proteomic analysis have identified alterations in the protein post-translational modifications (PTMs) profile of patients with AD lesions compared with non-lesional sites (Winget et al., 2016). Indicating that PTMs emerge key roles in AD development (Kim et al., 2016; Potaczek et al., 2017). In this review, we overview the roles of PTMs in the pathophysiology of AD, focusing on the known mechanisms and functions of PTMs with particular emphasis on their therapeutic potential.

## Post-Translational Modifications Relevance

PTMs are the chemical modifications of proteins following translation that confer functional diversity and maintain proteomes stability (Banerjee Mustafi et al., 2017). It can rapidly regulate a variety of biological functions (including cellular signaling, growth, survival, and proliferation) and modify inflammatory signaling pathways (Wu et al., 2017; Liu et al., 2019). Notably, aberrant PTMs may drive the development of inflammatory dermatitis with genetic predisposition, such as psoriasis and AD (Yi and McGee, 2021). Identifying the relationship between AD and PTMs could shed light on the disease pathogenesis and provide targets for the development of novel therapeutic and diagnostic tools. This review summarizes the seminal discoveries of six major PTMs (phosphorylation, acetylation, ubiquitination, SUMOylation, glycosylation, and glycation during the progression of AD and arouse in-depth research into the pathogenesis of disease.

## Clinical Relevance

Notably, AD ranks 15th among the largest nonfatal disease burdens worldwide, while its pathogenesis is multifactorial and unclear in most affected individuals (Laughter et al., 2021; Ständer, 2021). Moreover, PTMs show therapeutic potential for curbing inflammation and restoring barriers, which are closely linked to numerous skin and autoimmune disorders (Yang and Yan, 2022). Exploring the indexes of PTMs therefore yield novel therapeutic targets, predictive events, monitor trends, or prognostic indicators for the clinical outcomes of AD patients. Current PTMs-related agents are insufficient for implying clinical utility, and further mechanistic studies ought to be conducted.

## Pathological Manifestations in Atopic Dermatitis

### The Intricate Immune Responses in Atopic Dermatitis

The lesional skin has two major cell compartments: resident skin cells, which include keratinocytes (KCs), and infiltrating cells, which include inflammatory cells. Those cells function in synergy and produce numerous inflammatory mediators to recruit immune cells, activate intracellular signaling pathways, and stimulate adaptive immune responses, resulting in exacerbating pathogenicity in AD (Ständer, 2021). The elevated immunoglobulin E (IgE) level is a hallmark of AD, which causes a systemic inflammatory response; moreover, the activated cytokines and chemokines, in turn, are responsible for inducing IgE production (Gandhi et al., 2016). Once the epidermal barriers have been destroyed, alarm-type cytokines [such as IL-25, IL-31, IL-33, and thymus stromal lymphopoietin (TSLP)] could initiate innate immune components group 2 innate lymphoid cells (ILC2s), followed by the dysregulation of the T helper (T_H_) 2 response (Ständer, 2021). Skin DCs, which include epidermis-resident DCs, Langerhans cells, and dermal DCs, could simultaneously recognize allergens and microbes, enter the dermis, and migrate to the draining lymph node in response to specific antigens by promoting the polarization of T_H_ 2 cells (Callard and Harper, 2007; Novak, 2012).

**FIGURE 1:**
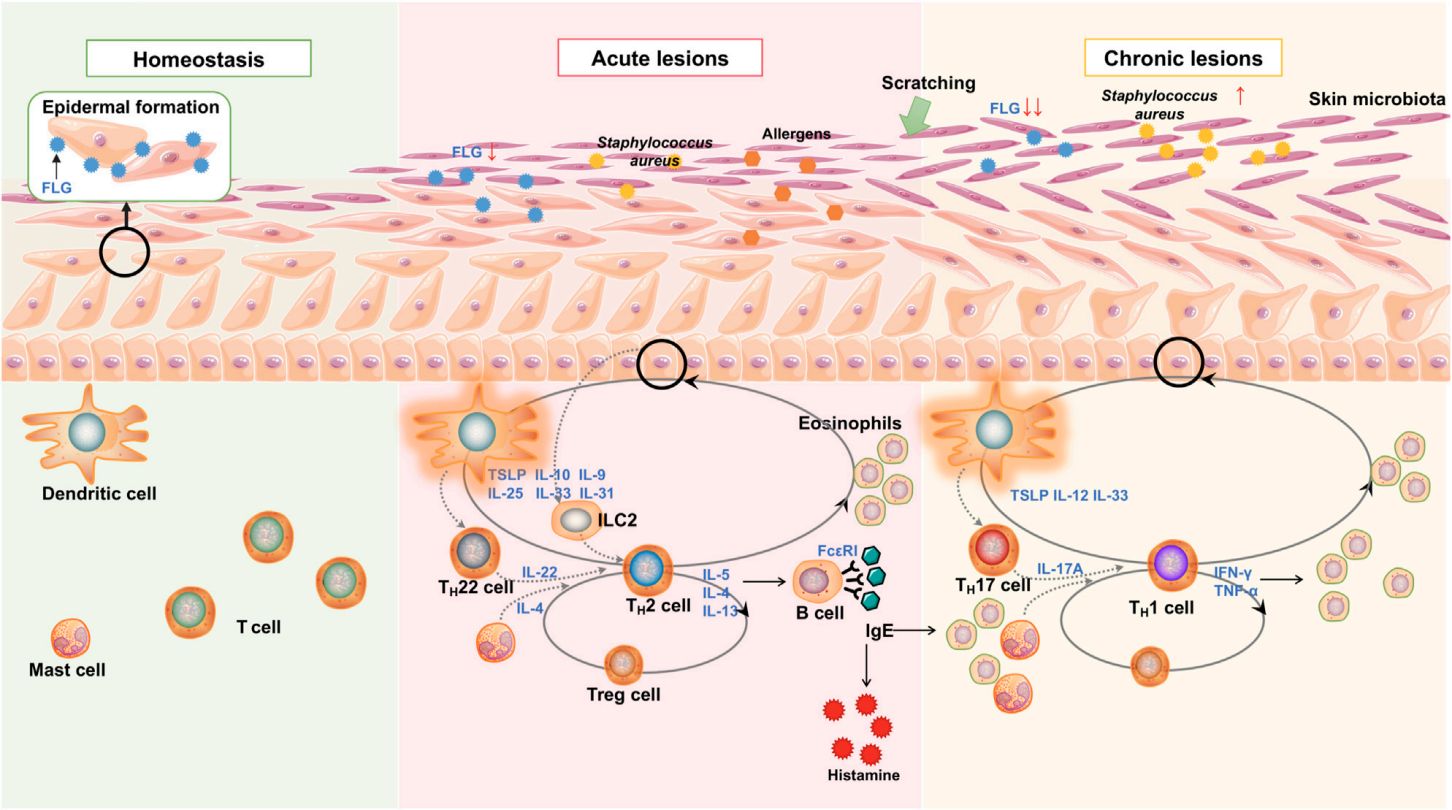
Main pathogenesis and mechanisms of AD. Epidermal barrier disruption mainly caused by mechanical scratch and aberrant inflammatory reactions. Reduced FLG contributes to inflammatory changes, while the releasing proinflammatory cytokines and chemokines result in further FLG deficiency. It is a positive feedback loop. LC and DC recognize the allergens and microbial components and stimulate adaptive immune responses—predominantly T_H_2/T_H_22 bias in the acute phase and T_H_1/T_H_17 bias in the chronic phase. The release of alarmins (including IL-10, IL-31, and TSLP) from epithelial cells promotes ILC2 induction as an innate immune response and aggravates T_H_2 immune response. T_H_2 cytokines IL-4, IL-5, and IL-13 recruit eosinophils and increase B cell IgE production during the acute phase. IL-22, produced by T_H_22, promotes T_H_2-type inflammation. In the chronic stage, T_H_1 and cytokines predominate in skin lesions, leading to further inflammation and epidermal hyperplasia. Inflammatory cytokines simultaneously impair the skin barrier by inhibiting barrier proteins and disrupting skin microbiota, thereby increasing the risk of *S. aureus* colonization.

Concerning adaptive immunity responses in AD, the presence of T_H_2/T_H_22 bias is shown in acute phases, while the T_H_1/T_H_17 bias is shown chronic phases (Patrick et al., 2021). In the acute phase, T_H_2 cells secreted cytokines (primarily IL-4, IL-5, and IL-13) that stimulate B cells to produce IgE antibodies and activate eosinophils, basophils, and mast cells in allergic responses; IL-5 is responsible for the trafficking and production of eosinophils *in vivo* (Gandhi et al., 2016). Activated ILC2s (expressing high levels of IL-5 and IL-13) could simultaneously facilitate T_H_2 differentiation and repress T_H_1 differentiation (Maggi et al., 2017), and IL-4 performs similar functions in AD pathogenesis (Murphy and Reiner, 2002; Chen et al., 2004; Lazarski et al., 2013). With the further identification of adaptive immunity in AD, acute lesions are always driven by the T_H_2/T_H_22 dominant allergic responses, while chronic lesions are driven by a T_H_1 response (Nograles et al., 2009; Lou et al., 2017). Local T_H_1 responses tend to induce KCs apoptosis through skin-infiltrating T cells (Grewe et al., 1998; Trautmann et al., 2000), and a correlation between T_H_17 cells and T_H_1 cells was found in the chronic phase of AD (Sugaya, 2020). The level of T regulatory cells (Tregs) is elevated during AD pathogenesis, which could suppress responses from allergen-specific T cells and participate in multi-directional immune reactions following T_H_2 and T_H_17 cells (Figure 1) (O’Shaughnessy et al., 2007; Agrawal et al., 2011; Jonak et al., 2011; Ma et al., 2014; Roesner et al., 2015; Niiyama et al., 2016).

### The Disruption of the Skin Barrier in Atopic Dermatitis

Cutaneous irritation and sensitization can give rise to barrier dysfunction and prolonged inflammation. Impaired epidermal barrier is indicated mainly by skin irritations, hypersensitivity, increased transepidermal water loss, decreased liposomes, and elevated pH (Ishikawa et al., 2010; Jungersted et al., 2010; Janssens et al., 2012). For instance, filaggrin (FLG, a major structural protein in the upper epidermis) assists in the formation of the epidermal barrier (O’Regan et al., 2008; Weidinger and Novak, 2016), which presents a loss-of-function in approximately 10%–40% of patients with AD (Rodríguez et al., 2009; Winge et al., 2011; Margolis et al., 2014; Feketea and Tsabouri, 2017). Besides, the breakdown of the skin barrier function is multi-factorial, involving disruption of the biological, immunological, and mechanical barriers (Weidinger and Novak, 2016), and its exacerbation is caused by infection, chronic irritation, inflammation, and immune dysregulation (Biniek et al., 2012; Yosipovitch et al., 2019). Notably, altered expressions of these epidermal proteins could favor T_H_2 dominance in T-cell differentiation (Figure 1) (Hönzke et al., 2016).

### The Dysbiosis of the Skin Microbiome in Atopic Dermatitis

Dysbiosis of the skin microbiome was detected in the dermis of AD patients, which contributes as a prominent environmental factor in the pathogenesis (Nakatsuji et al., 2016; Nakatsuji and Gallo, 2019). Specifically, the colonization of *Staphylococcus aureus* in the dermal lesions is associated with increased disease severity (Nakatsuji et al., 2016), which could exacerbate skin inflammation by activating lymphocytes and macrophages in adaptive and innate immune responses (Nakatsuji and Gallo, 2019). In addition, T_H_2 cytokines inhibit the generation of antimicrobial peptides of *S. aureus* in the skin and contribute to strenuous dysbiosis and infection during AD progression (Figure 1) (Howell et al., 2006; Nakatsuji and Gallo, 2019).

## Involvement of Post-Translational Modifications in ADS

PTMs are essential for regulating protein folding, stability, localization, and functional activities. With the deep exploration of proteomics, exploring the involvement of PTMs in AD pathogenesis could generate novel ideas for disease therapeutics, control, and prevention. Several types of PTMs including phosphorylation, acetylation, ubiquitination, SUMOylation, glycosylation and glycation were summarized under current research situation.

### Phosphorylation

Protein phosphorylation is an important modification for modulating protein stability, which occurs in almost one-third of the human proteome. The reversible process of phosphorylation/dephosphorylation is catalyzed by protein kinases and phosphatases, and is involved in cellular proliferation, growth, differentiation, and signal transduction (Krebs and Beavo, 1979). Phosphorylation frequently occurs at serine, threonine and tyrosine, which modulates various cellular signaling pathways and biological processes.

### Mitogen-Activated Protein Kinases

MAPKs, a cluster of serine/threonine kinases protein, could be activated by phosphorylation, which participant in cell proliferation, differentiation, survival and regulate pivotal signal transcription (Song et al., 2015; Park et al., 2020). Three major MAPKs have been identified, including p38, extracellular signal-regulated protein kinase (ERK), and c-Jun N-terminal kinases (JNK).

MAPKs play facilitatory roles in the susceptibility to inflammation, which could activate the TNF-α signaling cascades and energize the NF-κB signaling pathways (Giridharan and Srinivasan, 2018); in turn, inflammatory cytokines could promote MAPK phosphorylation and aggravate the secretion of those mediators in AD development (Hong et al., 2015; Ryu et al., 2015; Noh et al., 2016; Pinto et al., 2018; Naruke et al., 2021; Ogura et al., 2021). Specifically, higher FcεRI (a receptor of IgE, can be upregulated by p38 phosphorylation) is detected in peripheral blood monocytes of AD patients compared with normal individuals, which increases the susceptibility of microbial antigens and allergens (Song et al., 2015). Besides, FcεRI-bound IgE migrates to the lymph nodes and stimulates naive T cells to expand and trigger the T_H_2 immune response in AD pathogenesis (Figure 1) (Song et al., 2015). Increased IL-9 levels in AD lesions suggest the activation of ERK phosphorylation, which contributes to the spontaneous induced inflammation (Hong et al., 2015). Intelectin-1 (ITLN1, can induce ERK phosphorylation and help to amplify the T_H_2 responses) is overexpressed in AD skin lesions, accompanied by significant expression of allergen-induced TSLP, IL-33, and IL-25 (Tsuji et al., 2001; Yi et al., 2017). In AD therapies for the pediatric population, vitamin K2 exerts an anti-inflammatory effect by means of inhibiting the JNK signaling and ERK1/2 phosphorylation (Zhang et al., 2021). Above studies present that MAPKs phosphorylation is a crucial biologic activator in triggering immune responses and amplifying allergic inflammation in the pathogenesis of AD.

Consistent with the results in AD patients, an increasing level of phosphorylated ERK1/2 was found in the 2,4-dinitrochlorobenzene (DNCB)-induced mouse model (Choi et al., 2021). Deletion of *AnxA1* (an upstream regulator of ERK) leads to ERK hyperphosphorylation and aggravates local skin lesions with severe erythema, erosion, dryness, and epidermis thickening in the OVA-induced AD mice (Perretti and Gavins, 2003; Parisi et al., 2019). Alternatively, phosphorylated JNK and ERK inhibit the expression of FLG, which lead to the impaired epithelial integrity in AD mouse model (Cha et al., 2019a). Hence, we theorized that skin barrier homeostasis could be disrupted not only by phosphorylated MAPKs directly, but also by phosphorylation-activated inflammatory environment.

Based on the overexpressed ITLN1 in skin lesions of AD patients, further investigation confirmed that ITLN1 inhibitor could interfere with the phosphorylation of epidermal growth factor receptor (EGFR) and ERK and suppress the T_H_2 immune responses *in vitro* (Yi et al., 2017). High phosphorylated ERK1/2 was found in the TNF-α/IFN-γ induced HaCaT, which could activate the NF-κB and signal transducer and activator of transcription 1 (STAT1) signaling pathways and enhance inflammatory cytokines (Choi et al., 2021). Similar to other cytokine receptors, the activation of ERK phosphorylation requires calcium propagation; that is, the inhibition of calcium could, at least partially, disrupt ERK phosphorylation and ameliorate AD symptoms (Hong et al., 2015). IL-31, a relatively novel itch-relevant cytokine associated with T_H_2 cytokines, gives rise to calcium propagation and inflammation in primary human KCs *in vitro* (Lee et al., 2012). Moreover, phosphorylated JNK and ERK increases TSLP level in HaCaT (Jang et al., 2013), whereas the phosphorylation of p38 and ERK up-regulates IL-33 expression in IL-17A-induced normal human epidermal keratinocytes (NHEK) cells (Meephansan et al., 2013). Apart from proinflammatory properties, MAPKs phosphorylation is associated with fragile skin barrier as well. Hyperphosphorylated p38 can inhibit several junctional proteins (such as ZO-1 and RhoA) and destroy barrier function, and those barrier junctional proteins can be restored by inhibiting p38 kinase in NHEK cells (Jackson et al., 2011; Kanemaru et al., 2017). Furthermore, the inhibition of JNK could recover the reduction of FLG in TNF-α/IFN-γ induced HaCaT and block TNF-α-mediated inhibition of FLG and loricrin (LOR) in primary human KCs (Kim et al., 2011; Cha et al., 2019b).

In summary, MAPKs work as inflammatory amplification regulators and skin homeostasis destroyers in the pathogenesis of AD. The initial inflammatory responses activate MAPKs phosphorylation and the downstream factors, and then phosphorylated MAPKs urge the release of multiple pro-inflammatory factors, exacerbate the T_H_2 immune skew (Park et al., 2015; Choi et al., 2021), and involve in the regulation of skin barrier functions. Although there are no direct functional evidences of MAPKs necessity in AD pathogenesis, it demonstrated that MAPKs is associated with the disease attack and their inhibitors may provide new insights into the therapeutic drugs of AD treatment.

### AKT (Protein Kinase B)

AKT, also called protein kinase B, is a serine/threonine kinase phosphorylated by phosphoinositide-dependent kinase-1 and mammalian target of rapamycin (the mTORC1 inhibitor) complex2 (Laplante and Sabatini, 2012; Nitulescu et al., 2018). Phosphoinositide 3-kinase (PI_3_K) could activate AKT and its downstream mTOR pathways, and AKT phosphorylates downstream factors in the promotion of diverse cellular functions, including growth, proliferation, and metabolism (Liu et al., 2014; Huang et al., 2018). Recent research have demonstrated that the PI_3_K/AKT/mTOR pathway contributes to the development of hyperproliferative and inflammatory skin diseases *via* regulating KCs proliferation and immune responses (Mercurio et al., 2021).

The PI_3_K/AKT/mTOR pathway is activated abnormally in AD patients’ skin lesions and peripheral T cells, as well as in the skin of DNCB-induced and oxazolone-induced AD mouse models (Xiao et al., 2017; Hu et al., 2021; Zheng et al., 2022). LY294002 (an inhibitor of PI_3_K signaling) rescues claudin1 expression in AD mice *via* inhibiting AKT phosphorylation (Hu et al., 2021). Rapamycin proved to balance T_H_1 and T_H_2 immune responses *via* influencing cytokines production and suppressing serum IgE in an AD mouse model (Yang F. et al., 2014a); besides, it could increase the levels of FLG, LOR, and involucrin *in vitro* (Jia and Zeng, 2020).

AKT phosphorylation has been implicated in skin inflammation, which determines the activation of NF-κB cascade and participants in the hyperproliferation and expanded inflammation in human primary KCs (Lee et al., 2011; Madonna et al., 2012; Mercurio et al., 2021; Xiao et al., 2017). In parallel, normal AKT activity is required for epidermal barrier function, while the AKT/mTOR pathway controls the formation and maintenance of skin barrier (Naeem et al., 2017; Ding et al., 2020; Hu et al., 2021; Mercurio et al., 2021). Knock-down or deletion of AKT could decrease the expression of FLG and instigate hyperkeratosis both *in vivo* and *ex vivo* (Thrash et al., 2006; O’Shaughnessy et al., 2007; Naeem et al., 2017); the absence of AKT could impede KCs proliferation in HaCaT (Buerger et al., 2017). Further, mTORC2 and its related proteins control the expression of FLG through AKT in a phosphoinositide-dependent kinase 1-dependent manner (Naeem et al., 2017; Ding et al., 2020). Alternatively, LY294002 could attenuate the hyperproliferation of T cells and the secretion of pro-inflammatory cytokines in AD patient-derived T cells (Xiao et al., 2017).

In summary, these studies suggest that the inhibitor of AKT phosphorylation could act as putative candidates in inhibiting skin immunological reactions and restoring skin barrier dysfunction in AD.

### AMP-Activated Protein Kinase

AMPK is a serine/threonine kinase as well, whose early activation was evidenced by phosphorylation (Giansanti et al., 2020). It is always phosphorylated at threonine172 by liver kinase B1 and calcium/calmodulin-dependent protein kinase β (Shaw, 2009). AMPK is a principal cellular regulatory system, which maintains immune homeostasis and controls metabolic processes (Garcia and Shaw, 2017; Herzig and Shaw, 2018). Aberrant AMPK activation may contribute to diseases with abnormal proliferation of histiocytes, such as psoriasis and cancers (Garcin et al., 2015; Shen et al., 2021). Besides, AMPK is reported as a negative regulator of ERK signaling, as its deletion increased the ERK phosphorylation and the upstream pathway of ERK activation (Wu et al., 2013).

IL-37, an anti-inflammatory and immunosuppressive cytokine, counterbalances excess inflammation *via* activating AMPK signaling (Pan et al., 2020). Clinically, increased IL-37 expression was detectable in the skin lesions and serum of AD patients (Fujita et al., 2013), whereas IL-37 could attenuate inflammation symptoms, eliminate eosinophil infiltration, and increase Treg cells through the AMPK/mTOR signaling (Hou et al., 2020). Furthermore, AMPK could regulate the mTOR signaling to influence a range of cellular functions, including cell proliferation and metabolism (Shaw, 2009). The loss of AMPK performs direct effects on KC hyperproliferation and hyperactive mTOR signaling in a transgenic mice (Crane et al., 2021). Moreover, nicotine could decrease the risk of AD by inhibiting the expression of TSLP through a AMPK-mediated suppression of NF-κB signaling both *in vivo* and *in vitro* (Dong et al., 2016).

Above results reflect the mechanism of maintaining immunologic homeostasis and suppressing inflammation *via* AMPK phosphorylation in AD disorders; whereas additional molecular mechanisms are required to further clarify the relationship between AMPK and AD.

### Other Proteins

In addition to the aforementioned signaling molecules, we identified the other known proteins and their status associated with AD. Phosphorylation of ribosomal protein S6 is required for translation initiation and occurs on the serine residues of carboxyl-terminus, which is associated with the inflamed extent (Ruvinsky and Meyuhas, 2006; Ruf et al., 2014). Increased phosphorylated ribosomal protein S6 has been observed in epidermal lesions of AD patients, which is activated by the mTOR and Ras/ERK signaling (Ruf et al., 2014). Phosphorylated l-plastin (a leukocyte-specific actin-binding protein) increases in eosinophils from AD patients compared to healthy individuals (Noh et al., 2016), which could enhance inflammatory cell migration and aggravate phosphorylation in turn (Wabnitz et al., 2007; Pazdrak et al., 2011; Noh et al., 2016). Consistently, l-plastin phosphorylation could increase the migration of eosinophils (Pazdrak et al., 2011). Reflected that proteins phosphorylation play important roles in the migration of inflammatory cells and the amplification of immune responses. Heat shock proteins 27 (HSP27) can directly bind to AKT and result in increased phosphorylation of AKT and MAPK, which are required for KCs differentiation and epidermis formation (O’Shaughnessy et al., 2007; Jonak et al., 2011); further, elevated HSP27 levels were correlated with increased severity of the AD disease severity (Niiyama et al., 2016).

In general, phosphorylation is involved in the major pathogenesis of AD, including inflammation infiltration, immunization amplification, increased allergen sensitivity, and barrier maintenance. Multiple targets regulate distinct signaling pathways and provide diagnostic potential in AD management.

### Acetylation

Protein acetylation is one of the most common PTMs in which the acetyl group is introduced to a specific site on a polypeptide chain. Acetylation is a reversible process regulated by acetylase and deacetylase enzymes, involved in modulating chromatin structure, gene expression, and protein function (Verdin and Ott, 2015). The caveat here is that acetylation of histones (catalyzed by histone acetyltransferases) is associated with transcriptional activation; while histone deacetylation (catalyzed by histone deacetylases) is associated with transcriptional repression. Evidence suggests that histone acetylation and deacetylation are essential regulators of pro-inflammatory genes in allergic diseases, including histone deacetylase 3 (HDAC3), HDAC6, and sirtuin1 (SIRT1) (Alaskhar Alhamwe et al., 2018).

A clinical study confirmed that HDAC inhibitors could mitigate pruritus *via* decreasing IL-31 expression in peripheral blood from leukemic cutaneous T cell lymphoma patients (Cedeno-Laurent et al., 2015). As we know, to some extent, AD and T-cell mediated dermatitis share a similar underlying pathological mechanism. SIRT1 (an NAD-dependent protein deacetylase, which exists interdependent functions with HDAC6) plays a critical role in skin barrier maintenance, and is decreased in skin lesions of AD patients (Ming et al., 2015). Besides, belinostat (a histone deacetylase inhibitor) could restore skin barrier function and increase FLG expression in an *ex vivo* human skin culture model (Liew et al., 2020). Aryl hydrocarbon receptor nuclear translocator (ARNT; the loss-of-ARNT could increase HDAC levels) is up-regulated in skin lesions of AD patients compared to normal participants (Robertson et al., 2012; Kim et al., 2014; Hong et al., 2016). Results showed that some protein deacetylation could promote skin barrier renovation, while the relationship between barrier restoration and deacetylation degree remains obscure.

Furthermore, HDAC3 (a regulator of gene expression) is proven to mediate allergic inflammation, and its inhibitor could alleviate the skin inflammation of dinitrofluorbenzol (DNFB) -induced mice model (Kim et al., 2012). Similarly, HDAC6 (a regulator of immune responses) is a key point in regulating the activation and function of CD8^+^ T-cell inflammation responses in an AD mouse model (Tsuji et al., 2015) Phenylbutyrate (a kind of HDAC inhibitor) shows therapeutic effects on both acute and chronic skin inflammation *via* inhibiting local mast cells and activating Tregs in a DNFB-induced AD mouse model (Chung and Pui, 2011). Trichostatin A (TSA, another kind of HDAC inhibitor) can alleviate the DNFB-induced AD-like skin lesions in NC/Nga mice by exerting anti-inflammatory protective effects and increasing Treg cell population (Kim et al., 2010; Shi et al., 2012). In addition, SIRT1 is decreased in the cutaneous tissues of AD mouse model, and its modification provides relief from inflammation symptoms *via* suppressing the expressions of TSLP, cyclooxygenase-2, macrophage inflammatory protein 2, and C-X-C motif chemokine ligand 13 *in vivo* (Kwon et al., 2021). Previous studies have documented that SIRT1 plays an anti-inflammation role mainly by deacetylating NF-κB signaling (Yang et al., 2012; Kauppinen et al., 2013). On the other hand, mice lacking SIRT1 in KCs became susceptible to obtaining a fragile epithelial barrier with low expression of FLG (Ming et al., 2015). These findings suggest that HDAC inhibitors are found to be protective in various aspects of AD.

Notably, many studies focused on the use of HDAC inhibitors, and several active agents could work as HDAC inhibitors. Butyric acid and its derivatives, conducting as HDAC inhibitors, exert *in vitro* anti-inflammatory and antibacterial properties in HaCaT (Traisaeng et al., 2019). Similarly, sodium butyrate promotes cellular terminal differentiation and alleviates inflammatory responses induced by EGFR inhibition in NHEK cells (Leon Carrion et al., 2014). Propionate and valerate can also inhibit HDAC activity; they could increase pro-inflammatory factors secreted by KCs and decrease pro-inflammatory factors secreted by myeloid-derived immunocytes and ultimately exert a strong anti-inflammatory potential (Sanford et al., 2016). Given that those HDAC inhibitors should also be a group of anti-infective and anti-inflammatory proteins that play vital roles in inflammatory hyperproliferative diseases. Another, belinostat has been shown as a potential treatment for AD for its role in restoring FLG expression *via* inducing sustained miR-335 expression in N/TERT-1 cells (Liew et al., 2020). TSA decreases levels of FLG and LOR in N/TERT cells, resulting in worsen skin barrier (Robertson et al., 2012).

Overall, these findings suggest that acetylation modifications play an essential role in the skin barrier maintenance and inflammatory responses in AD progression. HDAC inhibitors emerging as promising therapeutic targets for AD treatment, while further in-depth studies are required.

### Ubiquitination

Ubiquitination is a dynamic and reversible PTM conserved in eukaryotic cells, which can tag proteins by proteasomes degradation (Feng et al., 2017). Proteasome is a part of the ubiquitin-proteasome system. The ubiquitin-proteasome system mediates the cellular polyubiquitination of substrate proteins and proteolytic degradation, including three types of enzymes: E1 ubiquitin-activating enzymes, E2 ubiquitin-conjugating enzymes, and E3 ubiquitin ligases. Deubiquitinating enzymes (DUBs) are responsible for specifically removing ubiquitin from ubiquitinated proteins and contributing to their stability (Ciechanover, 2015). Thus, ubiquitin could be re-used rather than degraded. Ubiquitinating and deubiquitinating can change intracellular homeostasis and modulate the cellular cycle, proliferation, and survival (Kwon and Ciechanover, 2017). Notably, current studies confirmed that E3 ubiquitin ligases and DUBs play roles in the AD pathophysiology (Fang et al., 2002; Loeser and Penninger, 2007a; Mohapatra et al., 2013a; G’Sell et al., 2015; Theivanthiran et al., 2015; Liu et al., 2017; Salva et al., 2017; Tang et al., 2018; Devos et al., 2019; Harirchian et al., 2019; Sundberg et al., 2020; Wang et al., 2021).

Genome-wide analysis found that AD pathogenesis involves a variety of gene expression abnormalities and ubiquitinated proteins (Acevedo et al., 2020). The tripartite motif 32 (Trim32, a member of the Trim E3-ubiquitin ligase family) is decreased in the skin lesions of AD patients, which implicates in inflammatory and immune processes (Guttman-Yassky et al., 2009; Liu et al., 2017). Sharpin (an adaptor protein for the linear ubiquitin chain assembly complex) is decreased in the lesions of AD patients, which appears as a potential anti-inflammatory candidate (Tang et al., 2018). Besides, casitas B-lineage lymphoma (c-CBL, a ring-type E3 ubiquitin ligase) presents the high expression level in the skin lesions from AD patients, which associated with T cell activation dysregulation and excess inflammatory infiltration (Loeser and Penninger, 2007b; Mohapatra et al., 2013b; Salva et al., 2017). Zinc finger protein A20 (A20, a ubiquitin-editing enzyme) is an endogenous anti-inflammatory factor related to NF-κB signaling, which is down-regulation in the epidermis from AD patients by transcriptome analysis (Devos et al., 2019; Mooney and Sahingur, 2021). Evidences above present a strong correlation between ubiquitinated proteins and AD mechanisms, involving genetic factors, inflammation extents, and immune dysregulation.

According to the down-regulation of Trim32 and Sharpin in AD patients, similar findings were observed that Trim32-deficient mice present T_H_2-biased inflammation spontaneously developed in imiquimod-induced psoriatic dermatitis (Liu et al., 2017; Wang et al., 2021), while KCs-specific Sharpin knockout mice developed more severe inflammatory AD lesions compared to normal ones (Sundberg et al., 2020). Transmembrane protein 79 (TMEM79, a predisposition gene for AD) can specifically inhibit ubiquitin-specific peptidase 8 deubiquitination, and its deficiency shows more susceptible to developing skin inflammation, compromised barrier function, and spontaneous dermatitis (Lim et al., 2013; Sasaki et al., 2013; Saunders et al., 2013; Chen et al., 2020). ITCH (an E3 ubiquitin ligase of the HECT family) inhibit p38α signaling by ubiquitylation of TGF-activated kinase 1–binding protein 1; hence suppressing skin inflammation *in vivo* (Theivanthiran et al., 2015). *Itch*-deficient mice exhibit T_H_2 inflammation and a scratching phenotype, and their lymphocytes exhibit a preference for T_H_2 differentiation, which is associated with the inhibition of JunB ubiquitylation caused by ITCH (Fang et al., 2002). Based on the anti-inflammatory properties of A20, additional experiments showed that *A20*-deficient mice developed T_H_2-biased immune responses, systemic pro-inflammatory changes, epidermal hyperplasia, and a disrupted skin barrier (Devos et al., 2019). Further indicated that deregulated ubiquitination events were closely related to T_H_2 cell differentiation, barrier reparation, and worsened inflammatory responses in AD pathogenesis.

Recent scRNA-seq data demonstrated that overexpressed A20 could suppress inflammatory transcripts induced by IL-17A in NHEK cells (Harirchian et al., 2019), indicating that A20 provides a potential AD therapeutic modality against inflammation. Reportedly, MID-1, a kind of E3 ubiquitin ligase, promotes T_H_2 type inflammation in allergic asthma (Collison et al., 2013). Nedd4 family interacting protein 2 might be a protective factor, and its absence inhibits the catalytic function of Nedd4-family E3 ubiquitin ligase and inappropriate T_H_2 responses during AD pathogenesis (O’Leary et al., 2016). Clinical and experimental data proposed that Sharpin is down-regulated in AD tissues, and Sharpin-silencing could increase FLG expression *in vitro*, implicating Sharpin as a novel mediator in both inflammation responses and barrier repair (Tang et al., 2018). Furthermore, many E3 ubiquitin ligases in regulating KCs proliferation and maintaining the epithelial tight junction function, such as Trim21 (Yang et al., 2018, 21), March-3 (Leclair et al., 2016, 3), Nedd4-1 (Yan et al., 2021, 1), Nedd4-2 (Raikwar et al., 2010), Chip (Löffek et al., 2010; Katagata, 2011), Otulin (Hoste et al., 2021; Schünke et al., 2021) and Trip (Almeida et al., 2011).

In conclusion, above studies highlight the critical role of ubiquitination in the initiation, progression and outcome of AD, and E3 ubiquitin ligases might be potential therapeutic targets for AD therapy.

### Small Ubiquitin-Like Modifier Ylation

SUMOylation is similar to ubiquitination and has approximately 11 kD proteins attached to lysine residues on target proteins covalently (Feligioni et al., 2009). SUMOylation is catalyzed by SUMO-specific enzymes of E1, E2, and E3, and regulates several functional properties, including chromatin organization, transcription, and DNA repair (Flotho and Melchior, 2013).

Increased miR-146a (which targets and represses SUMO1 directly) is found in the inflamed biopsy tissues and serum of AD patients, and its deficiency aggravates inflammation in MC903 (calcipotriol)-induced AD mice (Rebane et al., 2014; Yan et al., 2019a). Besides, protein inhibitor of activated STAT1 (PIAS1, a SUMO E3 ligase) is down-regulated in the peripheral blood mononuclear cells of AD dogs, which exhibits increasing IL-13 concentration and functional insufficiency of Tregs (Majewska et al., 2016; Koury et al., 2019). Several other studies noted that PIAS1 could restrict the differentiation of Tregs (Liu et al., 2010; Yang et al., 2013). It is suggested that miR-146a and PIAS1 might be therapeutic targets for AD management, and deciphering the SUMOylation effects will be required to investigate this further.

### Glycosylation

Glycosylation is a highly dynamic and reversible PTMs of proteins, which is catalyzed by a series of enzymes and adhered to abundant glycans. The biosynthesis of glycans is an intricate process requiring the coordinated action of multiple glycosyltransferases and glycosidases to synthesize discrete structures (Agrawal et al., 2017). Glycosylation has been shown to alter the functional activities of proteins and result in autoimmune disorders (Zhou et al., 2021); besides, impaired glycosylation can cause marked serum IgE elevations and severe inflammation (Yang et al., 2014b; Zhang et al., 2014). Several types of glycosylation, including O-linked GlcNAcylation and N-linked glycosylation, have been aroused increasing attention.

Reportedly, the deficit of phosphoglucomutase3 (a critical sugar nucleotide in glycosylation precursor synthesis) results in a prolonged inflammation in AD patients with increasing serum IgE elevations (Yang et al., 2014b; Zhang et al., 2014); its knockdown in T cells tends to produce excessive T_H_17 and T_H_2 cytokines (Zhang et al., 2014). Langerhans cells can identify and interact with *S. aureus* directly through conserving β-N-acetylglucosamine modifications on wall teichoic acid, indicating that β-N-acetylglucosamine is a key trigger in bacterial skin infection (van Dalen et al., 2019). The glycosylation of IgE is important to regulate allergic diseases, which contains seven asparagine N-linked glycosylation sites, providing a compelling diagnostic strategy for AD and other atopic diseases (Shade et al., 2020). These findings highlight the role of glycosylation in the genetic factors, external environment, and immune progression of AD.

### Glycation

Protein glycation is a common PTMs, where proteins, lipids, and nucleic acids react non-enzymatically leading to the formation of advanced glycation end products (AGEs) (Hanafy et al., 2021). AGEs exacerbate the inflammatory response through promoting the release of proinflammatory factors and the production of reactive oxygen species; therefore, the accumulation of AGEs is commonly associated with inflammatory and metabolic disorders (Botros et al., 2017; Papagrigoraki et al., 2017). Evidence showed that the increased exogenous AGEs exposure enhances the risk of developing AD and other atopic diseases (Smith et al., 2017).

A previous study found that urinary pentosidine, an AGE formed by sequential glycation and oxidation, tends to be higher in AD patients with acute exacerbation (Tsukahara et al., 2003). Correspondingly, the level of AGEs in corneocytes from AD patients is increasing along with lesion severity (Hong et al., 2020). Additionally, an increasing expression of receptor for AGEs (RAGE) is observed in AD-like mouse models, which results in the release of pro-inflammatory cytokines (Dumitriu et al., 2005; Karuppagounder et al., 2015; Wang et al., 2018). In sum, AGEs provide an attractive addition to novel diagnostic markers in AD, while further studies will be necessary to assess the therapeutic efficacy and safety of AGE-associated inhibitors.

## Crosstalk Among PTMS in Atopic Dermatitis

Remarkably, PTMs located within the same protein (especially on histones) can influence each other, and regulate the structure, activity and function of proteins (Narita et al., 2019). They function as molecular switches, and modify the interaction of proteins with DNA, lipids and other proteins (Venne et al., 2014; Narita et al., 2019). Emerging data indicates that there is significant regulatory crosstalk between PTMs during AD pathophysiology, in which the initial PTM serves as an active trigger for the addition or removal of a second PTM (detailed in Table 1) (Lin, 2002; Laarse et al., 2018).

**TABLE 1:** Crosstalk between post-translational modifications involved in AD.

Crosstalk	Forms	Main findings	Functions	References
Acetylation and Phosphorylation	Positive	ERK phosphorylation depends on HDAC6	Contributes to skin inflammation	Kwon et al. (2021)
	Positive	lysine acetylation of STAT proteins promotes phosphorylation STAT, HDAC inhibitors decrease p-STAT	Contributes to T_H_2 differentiation and pruritus	Krämer et al. (2009), Zhuang (2013), Rösler et al. (2018)
Phosphorylation and Ubiquitination	Positive	NF-κB activation depends on phosphorylation-induced IκB ubiquitination	Contributes to skin inflammation and innate immune response	Choi et al. (2018), Giridharan and Srinivasan (2018)
Ubiquitination and SUMOylation	Positive	Trim32 induces PIAS4 ubiquitination and decreases SUMOylation levels	Contributes to skin inflammation and T_H_2 differentiation	Albor et al. (2006), Liu et al. (2010), Samaka and Basha (2020)
Uiquitination and Acetylation	Positive	p62 inhibits HDAC6 and prolonged protein ubiquitination	Contributes to keratinocyte apoptosis	Hou et al. (2020)
		MARCH-1 target HDAC11 ubiquitination	Contributes to T_H_2 differentiation	Oh et al. (2013), Kishta et al. (2018)

Abbreviation: AD, atopic dermatitis; ERK, extracellular signal-regulated protein kinase; MARCH-1, membrane associated Ring-CH-1; Trim32, tripartite motif 32; TH2, T helper 2 cells; SUMO, small ubiquitin-like modifier; STAT, signal transducer and activator of transcription 1; PIAS4, protein inhibitor of activated STAT 4; HDAC, histone deacetylase.

Janus kinase (JAK)-STAT pathway play roles in AD pathogenesis by means of regulating T_H_2 differentiation, and its activation is caused by the phosphorylation of the two main proteins (JAK and STAT) (Szalus et al., 2020). Interestingly, the lysine-acetylation of STAT could activate STAT phosphorylation directly, except JAK-induced phosphorylation (Krämer et al., 2009; Zhuang, 2013). It is promising that HDAC inhibitors provide synergistic effects on JAK inhibitors in AD treatment (Quintás-Cardama et al., 2012; Rösler et al., 2018; Su et al., 2021).

Increased ERK phosphorylation has been found in an HDAC6-dependent manner in mast cells isolated from AD mice skin tissue (Kwon et al., 2021), suggesting that acetylation is vital for regulating ERK phosphorylation. A similar resolution has been postulated in heart failure (Habibian and Ferguson, 2018). Besides, the activation of NF-κB depends on IκB phosphorylation and subsequent IκB ubiquitin-dependent degradation (Choi et al., 2018; Giridharan and Srinivasan, 2018). Thus, inhibiting IκB protein degradation is one proven approach for reduce immune activation and inflammation in AD treatment.

PIAS4 (an E3-SUMOylation ligase with comparable functions to PIAS1) was previously identified as a significant E3-ubiquitin ligase Trim32 substrate, while the *Trim32*-deficiency in KCs could result in PIAS4 accumulation and increase global SUMOylation (Albor et al., 2006; Kudryashova et al., 2012). Besides, Trim32 activates NF-κB to induce KCs apoptosis and thus upregulates T_H_17 *versus* T_H_2 immunity, which explains the lower expression of Trim32 observed in AD patients (Albor et al., 2006; Liu et al., 2010; Samaka and Basha, 2020). Indicating that Trim32 may mediate inflammatory responses through SUMOylation and ubiquitination.

Furthermore, acetylation affects protein stability by suppressing ubiquitination and vice versa (Li et al., 2010; Incani et al., 2014; Shimizu et al., 2021). p62 (a ubiquitin binding protein) has a high ubiquitin-binding activity and facilitates protein ubiquitination and degradation (Boyault et al., 2006; Moscat and Diaz-Meco, 2009; Galindo-Moreno et al., 2017; Zientara-Rytter and Subramani, 2019), and it could maintain the autophagic process in homeostasis through inhibiting HDAC6 expression (Yan et al., 2013; Galindo-Moreno et al., 2017). On the other hand, membrane associated Ring-CH-1 (MARCH-1, an E3 ubiquitin ligase) could facilitate the expression of OX40L (a co-stimulatory molecule that could induce T_H_2 inflammation in AD) and decrease the amount of thymic Treg cells *via* increasing HDAC11 ubiquitination (Oh et al., 2013; Kishta et al., 2018; Castellanos et al., 2021; Furue and Furue, 2021). These findings suggest that E3 ligases could specifically target HDAC ubiquitination and thus enhance histone acetylation in AD pathophysiology (Scognamiglio et al., 2008).

While the mechanisms of PTMs crosstalk in AD have not been thoroughly investigated, they are also important. Of additional concern is the negative crosstalk between these two PTMs remains poorly understood (Venne et al., 2014; Mondal et al., 2021).

## Potential Clues for Atopic Dermatitis Diagnosis and Treatment

The prevalence of AD is increasing worldwide, although estimates in developed countries are stabilizing (Langan et al., 2020). Currently, topical corticosteroids are still the first-line therapy for AD (Cury Martins et al., 2015). If corticosteroids become ineffective or present adverse effects, treatment with phototherapy, systemic immunotherapy, and molecular targeted therapies (such as dupilumab, tralokinumab, baricitinib, and upadacitinib) would be used (Wollenberg et al., 2019; Blauvelt et al., 2021). Given the exceptional performance of biological agents in the treatment of autoimmune diseases, bio-therapeutics may be the future of moderate-to-severe AD treatment.

We emphasized that aberrant PTMs could trigger complex cascades of multi-cellular and multi-factorial pathways in AD pathophysiology, and the diagnostic and prognostic significance of PTMs should however be mentioned. Currently, dermal AGEs and urinary pentosidine have been used as biomarkers for early detection and prognosis estimation in AD (Tsukahara et al., 2003; Hong et al., 2020). With the introduction of skin autofluorescence in AGEs measurement, it raises the possibility of non-invasive tools in assessing both the disease severity and the comorbidity risks in the future (Ying et al., 2021). Besides, specific IgE glycosylation sites appear to be more sensitive biomarkers compared to serum IgE in the early differential diagnosis of atopic diseases (Shade et al., 2019; Shade et al., 2020). Hence, PTMs could work as potential biomarkers for diagnosis and prognosis of AD.

Furthermore, PTMs participate in AD pathophysiology *via* regulating related transcriptional factors, signaling molecules and proteins; targeting PTMs are prone to improve the synergistic effect of AD treatment. Growing evidence proves that PTMs are key effectors in skin inflammation and T_H_2 differentiation, which precisely improve the stability and activity of diverse signaling pathways including JAK/STAT, PI_3_K/AKT/mTOR, and NF-κB signalings (Liu et al., 2014; Choi et al., 2018; Giridharan and Srinivasan, 2018; Huang et al., 2018; Szalus et al., 2020). Besides, activating protein PTMs contributes to the maintenance of epidermal homeostasis, involved in regulating KC proliferation, sustaining tight junction function, and assisting barrier formation. To date, HDAC inhibitors (including vorinostat, romidepsin, belinostat, and panobinostat) have been approved by the FDA for the treatment of cutaneous T-cell lymphoma, peripheral T-cell lymphoma and multiple myeloma, whereas those diseases share similar pathological mechanisms with AD (Yi and McGee, 2021). Although there are no demonstrated PTM targeting approaches for AD treatment currently, some small molecule compounds (such as HADC inhibitors, MAPK inhibitors, and AKT inhibitors) modifying PTMs have shown therapeutic efficacy at the animal and cellular levels (Table 2). While none of these studies have explicitly focused on alleviating AD symptoms, the demonstrated PTMs-related biological effects both *in vivo* and *in vitro* are encouraging results that may bring PTMs-related therapeutics to the forefront in AD research.

**TABLE 2:** Potential therapeutic target in AD associated with protein post-translational modifications.

Modifiers	Agents	Targets	Functions	References
MAPK inhibitors	vitamin K2	JNK, ERK	Suppress skin inflammation; attenuate activated T-cell immunity	Zhang et al. (2021)
	Galactose	ITLN1	Interfere ERK phosphorylation; suppress T_H_2 immune responses	Yi et al. (2017)
	BTP2/SKF96365	STIM1	Suppress skin inflammation	Hong et al. (2015)
	SB202190	p38 MAPK	Repair skin barrier	Kanemaru et al. (2017)
	SP600125	JNK	Repair skin barrier	Cha et al. (2019a)
AKT inhibitors	LY294002	PI_3_K	Suppress T cell immune responses; inhibit serum IgE and skin inflammation; repair skin barrier	Xiao et al. (2017), Hu et al. (2021)
	Rapamycin	mTOR	Suppress TH2 immune responses; repair skin barrier	Yang et al. (2014a)
PKC inhibitor	4,5-bis (4-fluoroanilino)	PKCβII	Inhibit l-plastin phosphorylation	Pazdrak et al. (2011)
HDAC inhibitor	Butyric acid	Most HDACs, except Class IIB and III	Reduce *S. aureus* colonization; decrease pro-inflammatory interleukins	Davie (2003), Traisaeng et al. (2019)
	Phenylbutyrate	Most HDACs, except Class IIB and III	Inhibit local mast cells; activating Tregs	Chung and Pui (2011)
	Tubastatin A	HDAC 6	Rescue barrier dysfunction; inhibit skin inflammation; activating Tregs	Kwon et al. (2021)
	Belinostat	Class I, II	Rescue barrier dysfunction	Liew et al. (2020), Quah et al. (2021)
	Trichostatin A	Class I, II	Suppress T_H_2 immune response	Kim et al. (2010), Banerjee et al. (2012), Shi et al. (2012)
Glycan inhibitor	neuraminidase	sialic acid	Attenuate allergic response	Shade et al. (2020)

Abbreviations: AD, atopic dermatitis; AKT, protein kinase B; ERK, extracellular signal-regulated protein kinase; HDAC, histone deacetylase; ITLN1, intelectin-1; JNK, c-Jun N-terminal kinases; mTOR, mammalian target of rapamycin; PI3K, phosphoinositide 3-kinase; PKCβII, protein kinase C βII; *S. aureus*, *Staphylococcus aureus*; STIM, stromal interaction molecule 1; TH2, T helper 2 cells; Treg, T regulatory cells; MAPK, mitogen-activated protein kinases.

## Summary

**FIGURE 2:**
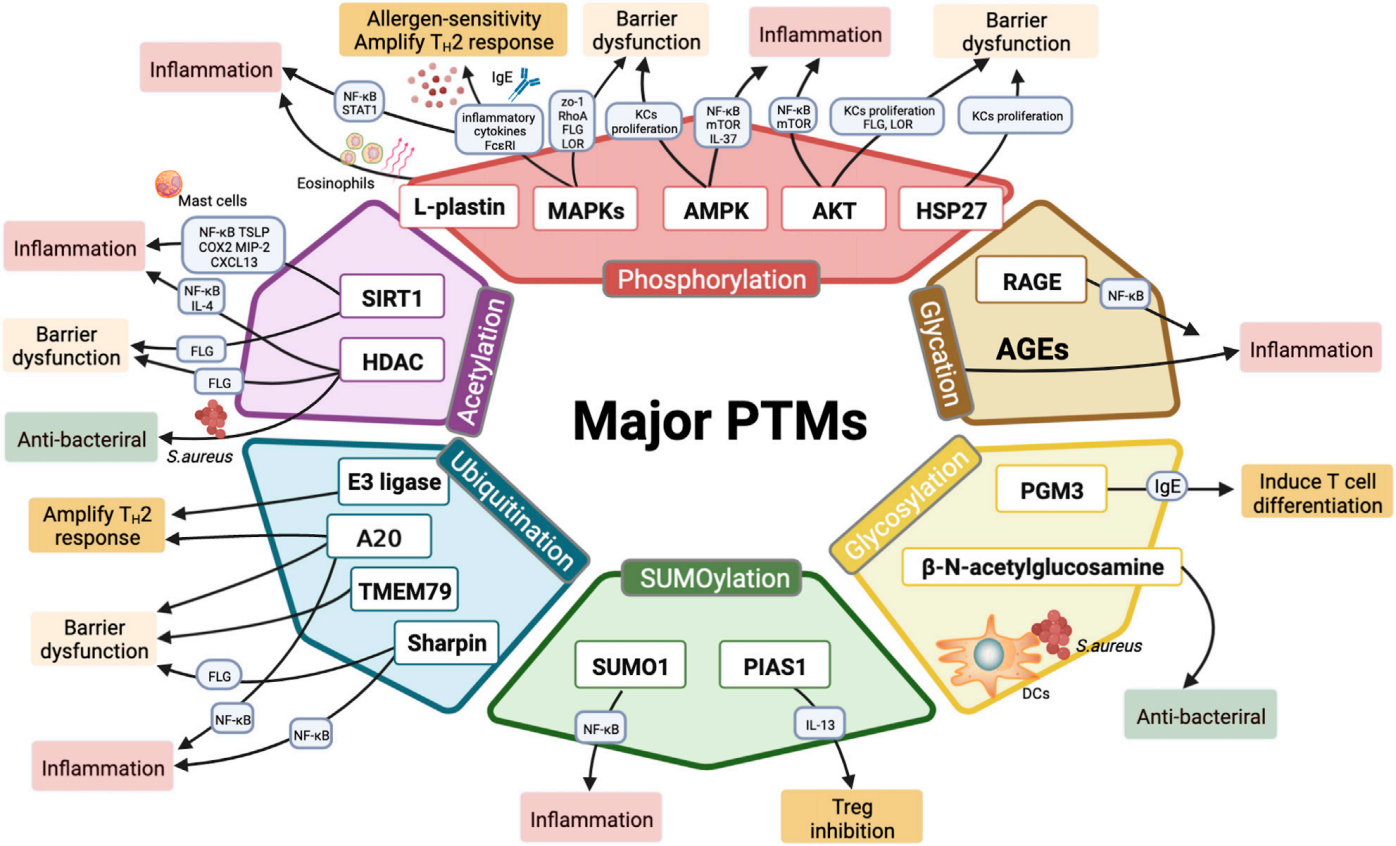
Major PTMs involved in the pathogenesis of AD. Six major classes of PTMs involved in AD include phosphorylation, acetylation, ubiquitination, SUMOylation, glycosylation, o-glycosylation, and glycation. Phosphorylation of PTM-related enzymes (MAPKs, AKT and AMPK) and l-plastin may regulate epidermal inflammation and T_H_2 immune response by modulating inflammatory cytokine secretion (IL-9, IL-25, IL-31, IL-33, TSLP, and IL-37), promoting eosinophils migration, and activating AD-related pathways (such as NF-κB, JAK-STAT, mTOR signaling). Moreover, MAPK p38 phosphorylation upregulates FcεRI and results in allergen-induced hypersensitivity; besides, MAPK phosphorylation inhibits epidermal proteins (FLG, LOR) and tight junction proteins (RhoA, ZO-1), thereby disrupting the barrier functions. Meanwhile, both AKT phosphorylation and AMPK phosphorylation are involved in epidermal barrier function. Phosphorylation of AKT and HSP27 are required for KCs differentiation and epidermis formation. Histone deacetylases (SIRT1, HDAC) play paramount importance in regulating inflammation and maintaining skin barrier functions. Additionally, HDAC inhibitors exhibit antibacterial properties in AD treatment. Multiple E3 ubiquitin ligases are linked to dysregulated T-cell activation and excessive inflammatory infiltration. Ubiquitination editing enzyme A20 maintains skin barrier hemostatic and alleviates inflammation. Other ubiquitination-related proteins (TMEM79, Sharpin) act as mediators in inflammation responses and barrier repair as well. MiR-146 targets SUMO1 to regulate epidermal inflammation, whereas PIAS1 restricts the differentiation of Tregs by elevating IL-13. PGM3 reduces inflammation by inhibiting IgE production and T cell differentiation. β-N-acetylglucosamine modifications promote the identification and the interaction of LCs with *S. aureus,* thereby triggering bacterial skin infections*.* AGEs are formed by glycation process and exacerbating inflammatory responses *via* releasing proinflammatory factors; RAGE, the receptor of AGEs, stimulates skin inflammation *via* activating NF-κB phosphorylation.

PTM is one of the later steps in protein biosynthesis, and modulating innate functions of proteins precisely. Characterized protein modifications could cause differentiation of pro-inflammatory T cells, altering inflammatory cytokines, maintaining the normal skin barrier function, increasing sensitivity to allergens, and triggering skin infections (summarized in Figure 2 and Table 3). Targeting these modifications could provide major benefits in AD management. Intriguingly, the interaction among single PTMs sites and synergistic effects among multiple PTMs through which could account for the incredibly complex links involved in the AD pathogenesis to some degree. Nowadays, there were no PTMs-mediated drugs for AD treatment in clinic, although several small molecule compounds function as HDAC inhibitors, MAPKs inhibitors and AKT inhibitors have become available. PTMs crosstalk can integrate diverse signals and vastly increase their regulatory potential in the course of AD disease, while lacks further experimental verification both *in vivo* and *in vitro*.

**TABLE 3:** Summary of major post-translational modifications involved in AD.

PTMs	Factors	Expression	Subjects	Process participation	Functions	Reference
Phosphorylation	ERK	↑	Human AD	Increase IL-9, IL-33, and TSLP; activate NK-κB and STAT1 signaling; inhibit FLG induction	Promote inflammation; amplify the T_H_2 responses; disrupt barrier	Jang et al. (2013), Meephansan et al. (2013), Hong et al. (2015), Yi et al. (2017), Cha et al. (2019b), Park et al. (2019), Choi et al. (2021), Zhang et al. (2021), Zeze et al. (2022)
			Animal: DNFB-induced mice, MC903-induced mice			
			NC/Nga mice			
			Cell: HaCaT, NHEK			
	p38	↑	Human AD	Upregulate FcεRI, IL-33; inhibit ZO-1 and RhoA	Increase antigen-sensitivity; trigger T_H_2 immune response; disrupt barrier	Meephansan et al. (2013), Song et al. (2015), Kanemaru et al. (2017)
			Cell: NHEK			
	JNK	↑	Human AD	Increase TSLP; inhibit FLG and LOR induction	Promote inflammation; disrupt barrier	Kim et al. (2011), Jang et al. (2013), Cha et al. (2019a), Zhang et al. (2021)
			Animal: DNCB-induced mice			
			Cell: HaCaT, NHEK			
	AKT	↑	Human AD	Increase inflammatory cytokines, reduce FLG, LOR, INV, claudin1	Promote skin inflammation; promote hyperproliferation; disrupt barrier	Yang et al. (2014b), Naeem et al. (2017), Xiao et al. (2017), Jia and Zeng (2020), Hu et al. (2021), Mercurio et al. (2021)
			Animal: DfE-induced mice, oxazolone-induce mice			
			Cell: HaCaT, rat epidermal keratinocytes			
	AMPK	↑	Animal: MC903-induced mice	Inhibit NK-κB and mTOR signaling; suppress KC hyperproliferation	Suppress skin inflammation; suppress hyperproliferation	Hou et al. (2020), Crane et al. (2021), Dong et al. (2022)
			Cell: co-culture of primary human dermal fibroblasts and eosinophils, PAM212 cells			
	Ribosomal protein S6	↑	Human AD	Increase inflammatory cytokines and KCs differentiation	Promote skin inflammation	Ruvinsky and Meyuhas (2006), Ruf et al. (2014)
	L-plastin	↑	Human AD	Enhance eosinophil migration	Promote skin inflammation	Pazdrak et al. (2011), Noh et al. (2016)
			Cell: EoL-1 cells			
	HSP 27	↑	Human AD	Promote KC differentiation and FLG processing	Improve barrier formation	(O’Shaughnessy et al., 2007; Jonak et al., 2011; Niiyama et al., 2016)
Acetylation	HDAC3	↑	Animal: DNFB-induced mice	Active MCP1	Promote skin inflammation	Kim et al. (2012)
			Cell: RBL2H3 cells, mast cell			
	HDAC6	↑	Animal: DNCB-induced mice, TNCB-induced mice	Increase CD8^+^ T cell inflammation	Promote skin inflammation	Tsuji et al. (2015), Kwon et al. (2021)
			Cell: HaCaT, co-culture of mouse skin dermal fibroblast cells and mast cells			
	SIRT1	↓	Human AD	Suppress inflammatory cytokines; deacetylate NF-κB; promote FLG expression	Suppress skin inflammation; improve barrier dysfunction	Kauppinen et al. (2013), Ming et al. (2015), Lee et al. (2016), Kwon et al. (2021)
			Animal: DNCB-induced mice, ovalbumin			
			-induced mice			
			Cell: HaCaT			
Ubiquitination	TRIM32	↓	Human AD	Ubiquitinate PKCζ and inactivate NF-κB and TLR signaling	Restrain T_H_2 differentiation	Liu et al. (2017), Wang et al. (2021)
			Animal: MC903-induced mice			
	ITCH	↓	Animal: itchy mice	Ubiquitinate Tab1 and JunB; inhibit p38α phosphorylation	Restrain T_H_2 differentiation	(Fang et al., 2002; Theivanthiran et al., 2015)
	TMEM79	↓	Animal: flaky tail mice	Inhibit Wnt/Frizzled signaling	Maintain skin barrier integrity	(Sasaki et al., 2013; Saunders et al., 2013; Chen et al., 2020)
	c-CBL	↑	Human AD	Ubiquitinate PTKs; inhibit TCR signal transduction	Promote T-cell apoptosis	Loeser and Penninger (2007a), Mohapatra et al. (2013a), Salva et al. (2017)
			Cell: human CTCL cells			
	Sharpin	↓	Human AD	Inactivate IL-33/ST2, NF-κB, and JAK/STAT signaling; suppress FLG expression	Restrain T_H_2 immune Response improve barrier dysfunction	(Tang et al., 2018; Sundberg et al., 2020)
			Animal: mouse			
			Cell: HaCaT			
	A20	↓	Human AD	Ubiquitinate IκBα; inhibit NF-κB activation	Suppress skin inflammation; restrain T_H_2 differentiation	G’Sell et al. (2015), Devos et al. (2019), Harirchian et al. (2019)
			Animal: mouse			
			Cell: NHEK			
SUMOylation	SUMO1	↓	Human AD	Targeted by miRNA-146a	Promote skin inflammation	Yan et al. (2019b)
			Animal: DNCB-induced mice			
			Cell: 293T cells			
	PIAS1	↓	Animal: AD dogs	Restrict Tregs differentiation	Suppress skin inflammation	Majewska et al. (2016)
Glycosylation	PGM3	↓	Human	Decrease IgE levels and T_H_2/T_H_17 cytokines	Suppress allergic response	Zhang et al. (2014)
	β-N-acetylglucosamine	↑	Animal: epicutaneous infection mice	Trigger *Staphylococcus aureus*	Induce skin inflammation	van Dalen et al. (2019)
			Cell: MUTZ-3-derived LCs, primary human LCs			
Glycation	RAGE	↑	Animal: DfE- induced mice, DNCB-induced mice	Activate NF-κB phosphorylation	Promote skin inflammation	Karuppagounder et al. (2015), Wang et al. (2018)

Abbreviations: A20, Zinc finger protein A20; AD, atopic dermatitis; AGE, advanced glycation end products; AKT, protein kinase B; AMPK, AMP-activated protein kinase; c-CBL, casitas B-lineage lymphoma; CTCL, cutaneous T-cell lymphoma; DfE, dermatophagoides farina extract; DNCB, 2,4-dinitrochlorobenzene; ERK, extracellular signal-regulated protein kinase; FLG, filaggrin; HDAC, histone deacetylase; Hsp, heat shock proteins 27; IL, interleukin; INV, involucrin; JAK, janus kinase; JNK, c-Jun N-terminal kinases; KC, keratinocytes; LC, langerhans cells; LOR, loricrin; MC903, calcipotriol; MCP1, monocyte chemoattractant protein; NHEL, normal human epidermal keratinocyte; SIRT1, sirtuin1; PGM3, phosphoglucomutase3; PIAS1, protein inhibitor of activated STAT1; PKCζ, protein kinase C zeta; PTK, protein tyrosine kinase; RAGE, receptor for AGEs; STAT, signal transducer and activator of transcription; STAT, signal transducer and activator of transcription; SUMO, small ubiquitin like modifier; Tab1, TGF-β activated kinase 1; T_H_2, T helper 2 cell; TNCB, 2, 4, 6-trinitrochlorobenzene; TLR, toll-like receptor; TMEM79, transmembrane protein 79; Trim32, tripartite motif 32; TSLP, thymic stromal lymphopoietin.

This review provides novel insights regarding the pathogenesis of AD and the development of PTMs-based strategies for inflammatory and allergic diseases therapy. However, the identification of PTMs-related regulators in AD remains in its infancy. Based on advanced proteomics techniques, the intensive studies of PTMs may open up new avenues for the evaluation criteria of AD. We look forward that PTMs-based diagnosis, monitoring and therapeutic approaches will be prevalent in AD patients and play major roles in the future.

## Abbreviations

AD, atopic dermatitis; DC, dendritic cell; FLG, filaggrin; IFN-γ, interferon gamma; IgE, immunoglobulin E; IL, interleukin; ILC2, type 2 innate lymphoid cells; LC, langerhans cells; *S. aureus*, *Staphylococcus aureus*; T_H_, T helper; TNF-α, tumor necrosis factor alpha; TSLP, thymic stromal lymphopoietin; KC, keratinocytes; A20, zinc finger protein A20; AD, atopic dermatitis; AGE, advanced glycation end products; AMPK, AMP-activated protein kinase; FLG, filaggrin; LOR, loricrin; HDAC, histone deacetylase; HSP27, hot shock protein 27; IL, interleukin; KC, keratinocytes; LC, langerhans cells; MAPK, mitogen-activated protein kinases; PGM3, phosphoglucomutase3; PIAS1, protein inhibitor of activated STAT1; RAGE, receptor for AGEs; *S. aureus*, *Staphylococcus aureus*; SIRT1, sirtuin1; STAT, signal transducer and activator of transcription; SUMO1, small ubiquitin like modifier 1; TH2, T helper 2 cells; TMEM79, transmembrane protein 79; TSLP, thymic stromal lymphopoietin; PTM, post-translational modifications.
